# Identification of Key Functional Gene Signatures Indicative of Dedifferentiation in Papillary Thyroid Cancer

**DOI:** 10.3389/fonc.2021.641851

**Published:** 2021-04-28

**Authors:** Weibo Xu, Cuiwei Li, Ben Ma, Zhongwu Lu, Yuchen Wang, Hongyi Jiang, Yi Luo, Yichen Yang, Xiao Wang, Tian Liao, Qinghai Ji, Yu Wang, Wenjun Wei

**Affiliations:** ^1^Department of Head and Neck Surgery, Fudan University Shanghai Cancer Center, Shanghai, China; ^2^Department of Oncology, Shanghai Medical College, Fudan University, Shanghai, China; ^3^CAS Key Laboratory of Nutrition, Metabolism and Food Safety, Shanghai Institute of Nutrition and Health, Shanghai Institutes for Biological Sciences, University of Chinese Academy of Sciences, Chinese Academy of Sciences, Shanghai, China

**Keywords:** papillary thyroid cancer, dedifferentiation, functional grouping, gene signature, LASSO, WGCNA

## Abstract

**Background:** Differentiated thyroid cancer (DTC) is the most common type of thyroid cancer. Many of them can relapse to dedifferentiated thyroid cancer (DDTC) and exhibit different gene expression profiles. The underlying mechanism of dedifferentiation and the involved genes or pathways remained to be investigated.

**Methods:** A discovery cohort obtained from patients who received surgical resection in the Fudan University Shanghai Cancer Center (FUSCC) and two validation cohorts derived from Gene Expression Omnibus (GEO) database were used to screen out differentially expressed genes in the dedifferentiation process. Weighted gene co-expression network analysis (WGCNA) was constructed to identify modules highly related to differentiation. Gene Set Enrichment Analysis (GSEA) was used to identify pathways related to differentiation, and all differentially expressed genes were grouped by function based on the GSEA and literature reviewing data. Least absolute shrinkage and selection operator (LASSO) regression analysis was used to control the number of variables in each group. Next, we used logistic regression to build a gene signature in each group to indicate differentiation status, and we computed receiver operating characteristic (ROC) curve to evaluate the indicative performance of each signature.

**Results:** A total of 307 upregulated and 313 downregulated genes in poorly differentiated thyroid cancer (PDTC) compared with papillary thyroid cancer (PTC) and normal thyroid (NT) were screened out in FUSCC cohort and validated in two GEO cohorts. WGCNA of 620 differential genes yielded the seven core genes with the highest correlation with thyroid differentiation score (TDS). Furthermore, 395 genes significantly correlated with TDS in univariate logistic regression analysis were divided into 11 groups. The areas under the ROC curve (AUCs) of the gene signature of group transcription and epigenetic modification, signal and substance transport, extracellular matrix (ECM), and metabolism in the training set [The Cancer Genome Atlas (TCGA) cohort] and validation set (combined GEO cohort) were both >0.75. The gene signature based on group transcription and epigenetic modification, cilia formation and movement, and proliferation can reflect the patient's disease recurrence state.

**Conclusion:** The dedifferentiation of DTC is affected by a variety of mechanisms including many genes. The gene signature of group transcription and epigenetic modification, signal and substance transport, ECM, and metabolism can be used as biomarkers for DDTC.

## Introduction

Papillary thyroid cancer (PTC) is the most common type of thyroid cancer, and the majority of PTCs exhibit a relatively good prognosis (1, 2). However, it has been observed recently that some PTCs may dedifferentiate in some situations. When PTC appears to be dedifferentiated, its prognosis becomes very poor, and conventional surgical treatment cannot achieve good therapeutic effects. Such patients often experience relapse or metastasis in a short period of time (3–5). At present, the treatment methods for poorly differentiated thyroid cancer (PDTC) and anaplastic thyroid cancer (ATC) are limited, and their progression mechanism is still unclear.

It has been shown that many ATC and PDTC result from dedifferentiation of DTC (6). In addition, some genetic abnormalities such as *TERT* and *TP53* mutation may play an important role (7, 8). A considerable number of studies have shown that occurrence and development of PDTC and ATC are closely related to immune microenvironment and epigenetic changes (9–12). Our previous study also revealed that some genes may have a significant impact on the initiation and progression of dedifferentiated thyroid cancer (DDTC) through metabolism-related pathways (13). However, considering that dedifferentiation of DTC is accompanied by a great increase in the degree of malignancy, it is likely that dedifferentiation must involve more than one mechanism. The mechanisms of how genes affect DTC dedifferentiation remain to be studied.

This study was oriented toward mining out differentially expressed genes among PDTC, PTC, and normal thyroid (NT) at the level of transcriptome and then classifying them into different groups based on their biological functions to explore possible dedifferentiation-related processes. We expect that our findings could provide a plausible basis for further study of PDTC, thereby helping to indicate prognosis and development of PTC and exploring the possibility of reversing dedifferentiation or re-differentiation.

## Methods

### Sample Collection

Six NT, five PTC, and five PDTC specimens were obtained from eight patients who underwent surgical management in the Fudan University Shanghai Cancer Center (FUSCC) (Supplementary Table 1). The information of the eight patients and 16 samples was described in our previous study (13). These 16 samples were included in a discovery cohort and used for high-throughput RNA sequencing (RNA-seq) to identify differentially expressed genes. Written informed consent was obtained from each patient before his/her specimens were used in this study, and the study was approved by the Medical Ethics Committee of the FUSCC. All procedures performed in this study were in accordance with the Declaration of Helsinki.

### RNA-Seq Analysis

Total RNA was extracted from all samples using TRIzol reagent (Life Technologies, Carlsbad, CA). We used RiboMinus eukaryote kit (Qiagen, Valencia, CA) to remove ribosomal RNA of total RNA (~3 mg) before RNA-seq libraries construction. Strand-specific RNA-Seq libraries were prepared using the Illumina workflow (New England BioLabs, Beverly, MA). Next, the samples were fragmented, reverse-transcribed, and ligated to Illumina adaptors. We purified the ligated cDNA products to remove second-strand cDNA. After 13–15 cycles of amplification, libraries were controlled for quality and quantified using with an Agilent 2100 Bioanalyzer (Agilent Technologies, Santa Clara, CA) and sequenced by a HiSeq 2000 sequencing system (Illumina, San Diego, CA) on a 100-bp paired-end run. Clustal Omega was used for sequence alignment. Human genome version GRCh38.100 was used throughout. RNA-seq data were normalized by fragments per kilobase per million (FPKM). Significant differences were determined by Limma package (version 3.11; https://bioconductor.org/packages/limma/).

### The Cancer Genome Atlas and Gene Expression Omnibus Cohorts

The Cancer Genome Atlas (TCGA) mRNA expression data (FPKM) was sourced from the University of California Santa Cruz (UCSC) Cancer Browser (14). The raw data of Gene Expression Omnibus (GEO) combined GEO cohort including the GSE29265 cohort (20 NTs, 20 PTCs, and 9 ATCs) (15), GSE33630 (16), GSE53157 (17), GSE65144 (18), and GSE76039 (19) were obtained from the GEO database (20, 21) and were background adjusted and normalized. The ComBat method was performed to remove batch effects by the R package “sva.” According to the annotation file, probes were matched with gene symbol, and probes that were not matched to gene symbol were deleted. When more than one probe matched the same gene symbol, average value was calculated as the final expression value. We performed a correlation analysis on expression matrix of PDTC and ATC samples in the combined GEO cohort, and we found that PDTC and ATC are highly correlated (Supplementary Figure 1). Therefore, we used the GSE29265 cohort and GSE33630 cohort as verification cohorts to verify the effectiveness of gene signatures. All patients were staged by the 8th edition of the TNM staging system published by the American Joint Committee on Cancer. Only samples for which all clinical data and thyroid differentiation score (TDS) could be obtained were included in the analysis.

### Gene Function Annotation and Interaction Analysis

Gene Set Enrichment Analysis (GSEA) was performed using GSEA software. C5 collection [Gene Ontology (GO)] was utilized to identify GO terms that were differentially regulated in different comparisons. GO and Kyoto Encyclopedia of Genes and Genomes (KEGG) analysis were performed by DAVID online function annotation tool (22, 23) to classify differentially expressed genes into functional categories. The enriched group was ranked by *p-*value (*p* < 0.05) and false discovery rate (FDR < 0.25). The GeneMANIA prediction server was used for interaction analysis between genes (24).

### Weighted Gene Co-expression Network Analysis

Weighted gene co-expression network analysis (WGCNA) was performed by the R WGCNA package (25) (v1.66) to identify TDS-related modules and their gene members. The modules were identified by Dynamic Hybrid Tree Cut algorithm. We chose the yellow module with the highest correlation coefficient to screen out hub genes.

### Statistical Analysis

A comparison of categorical variables was performed using chi-square test and Fisher's exact test. Descriptive statistics are presented in the tables. TDS was proposed by TCGA project. It summarizes the expression of 16 genes (*DIO1, DIO2, DUOX1, DUOX2, FOXE1, GLIS3, NKXX2-1, PAX8, SLC26A4, SLC5A5, SLC5A8, TG, THRA, THRB, TPO*, and *TSHR*) related to thyroid metabolism and function, which can be an index to determine the degree of thyroid-specific differentiation. We have eliminated these genes in subsequent calculations to avoid bias (26). According to median of TDS, TCGA dataset was divided into two groups: highly differentiated and poorly differentiated. Least absolute shrinkage and selection operator (LASSO) regression was performed on each function group to reduce the number of variables. Multivariate logistic regression was used to determine genes to be finally included in the signature; expression levels of the differential genes as signatures in each patient were integrated into a risk score fitted by logistic regression (Table 1). Non-parametric receiver operating characteristic (ROC) analysis was performed for each signature, and we calculated area under the ROC curve (AUC) to test its indicative power for dedifferentiation. The Kaplan–Meier method was used to construct the disease-free survival (DFS) curve. Two-side *p* < 0.05 was considered statistically significant. Statistical analysis and visualization were carried out through SPSS (v25.0; IBM Corporation, Armonk, NY) and R (v3.6.3; R Foundation for Statistical Computing, Vienna, Austria) software.

**Table 1:** Function based differential gene grouping.

**Group**	**Genes**
Cell differentiation and rhythm	*CIPC, HHEX, NFIA, TCTN1, ARNTL2*
Substance and signal transduction	*ADCY9, ANK3, BMPR1A, CACNA1I, CHD6, CIRBP, CLIC3, DOP1A, DYNLL2, ERLIN2, FZD5, GPX3, ILDR1, KCNJ16, KCNQ1, KLHDC1, LMBRD2, NEBL, OBSL1, PCYOX1, PEBP1, PLLP, RAB30, RALGPS1, RIC3, SEC11C, SELENBP1, SLC20A2, SLC22A17, SLC48A1, TEF, TMEM50B, TOM1L2, TXNL1, AQP9, GAS2L3, GNA12, GPR173, KCNK6, KPNA2, MSR1, MYO1F, PPP1R18, PSTPIP2, SLC36A1, WASF1*
Transcription and epigenetic modification	*PRDM16, RORA, RORC, SALL2, SECISBP2L, SMAD6, SP4, TBX3, TPMT, ZBTB4, ZNF154, ZNF19, ZNF208, ZNF471, ZSCAN18*
Protein modification	*PTPN21, RNF146, RNF170, RNF180, USP51, USP54, ZNF10*
Cilia formation and movement	*BBOF1, BBS1, CFAP70, DNAH7, IQCA1, MAATS1, PIFO, TAPT1, TEKT2*
Extracellular matrix	*ADAM19, ANGPTL2, COLGALT1, PDGFA, RCN3, S100A3, SERPINH1, SH2D2A, SH3PXD2A, SLC8A1, SRPX2, TNC, TRPV2, AMIGO1, CPQ, CRHBP, NPNT, PARM1, PEF1, RGN, STX12*
Apoptosis	*ARL4C, BRIP1, EME1, EXO1, FANCI, FEN1, FHL3, GNA15, LMNB1, LMNB2, MAP4K4, MCM10, MELK, RAD51, SLC20A1, SPOCD1, TOP2A, TPX2, AKTIP, ARHGEF5, BCL2, BEX4, BTG2, C2orf40, CDS1, GABARAPL2, GPRASP1, LAMTOR3, MPPE1, STK33, TENT5C, TERF2IP, TMEM192,*
Proliferation	*ANLN, ASF1B, ASPM, AURKB, BUB1, CDC45, CDCA5, CDCA8, CDK2, CDT1, CENPF, CENPI, CENPL, CENPW, CEP55, CHEK1, CHTF18, CLIP2, DIAPH3, DNMT1, DTL, E2F1, E2F7, E2F8, ECT2, FOXM1, GINS1, GINS4, GTSE1, HASPIN, HJURP, IQGAP3, KIF11, KIF14, KIF23, KIF2C, KIF4A, MAD2L1, MARVELD1, MCM2, MCM4, MCM5, MIS18A, MKI67, NCAPG, NCAPG2, NCAPH, NDC80, NEK6, NTMT1, NUF2, NUSAP1, ORC1, ORC6, PDCD11, PHF19, PIMREG, PPP1R14B, PRC1, RACGAP1, RNASEH2A, SCO2, SGO1, SKA3, SMC4, SNRPB, SPC24, TCF19, TRIO, TRIP13, TUBA1B, TUBB6, UBE2C, UBE2T, UHRF1, WDHD1, ZWILCH, ZWINT, NAP1L5, NEK11, RCBTB1, RPRD2, STRBP, TMEM30B, VEZT*
Invasion	*ANPEP, COL1A2, COL7A1, CORO1C, CTHRC1, EZH2, FMNL1, GREM1, LGALS1, NHSL1, PLAUR, PLXNA1, TPBG, TPM4, TWIST1, CD164, DMTN, EPB41L4B, EPB41L5, HOOK2, ID4, MARVELD2, MPP5, OCLN, PIK3CB, RBM47, RUFY3, SFTA3, SLIT3, ZFP3*
Metabolism	*ACOT7, ADAMTSL1, ARSI, B4GALT2, ENO1, GALNT6, HK3, KIF20A, MTHFD2, PKM, PLPP4, PYGL, RALA, RRM2, SLC7A5, TK1, TYMS, UGCG, ACE2, ACOX2, ADHFE1, AK8, AK9, ALDH5A1, ALDH9A1, CCDC28A, CERS4, CHPT1, CROT, CYP2C8, ENOSF1, EPHX2, EPM2AIP1, ETFRF1, FAM174B, GKAP1, GPD1L, HSD17B8, IDNK, INPP5J, ITM2B, IYD, LHPP, LPCAT2, MAN1C1, MDH1B, MTMR10, NAPEPLD, NME5, NT5C2, PDE1A, PLPP3, RALGAPA2, SLC16A11, SLC25A23, SLC25A4, SORD, ST3GAL1, ST6GAL2, TM7SF2*
Immunity	*ADGRE2, C5AR1, CCR1, CD276, CD300A, CEACAM4, CSF1R, CXCL1, CXCL5, ELF4, FCGR2A, FCGR2B, FPR2, LILRB2, MICB, NCF2, NFKBIE, NLRC4, NOD2, OSCAR, PSMD2, RAP2B, RELB, SECTM1, SIGLEC1, SIRPA, SIRPB2, SLAMF8, SPHK1, TNFAIP6, VSIG4, BMP7, CRBN, DUOXA1, METTL7A, PBXIP1, PRKCQ, SASH1, TRIM2, TXNDC11*
Ungrouped	*C12orf75, CMSS1, C11orf71, C15orf56, C16orf46, C1orf210, CCDC191, CCDC85A, ZNF273, ZNF334, ZNF415, ZNF483, ZNF518B, ZNF585B, ZNF626, ZNF680, ZNF763, CYB5D1, ERMP1, FAM189A2, FAM8A1, FBXO16, IGSF22, IQCK, KIAA1671, KLHDC2, KLHL14, LNX1, LYRM9, PLEKHB1, PLEKHH1, PPIL6, PPP1R21, PXK, SPATA6L, THAP6, TMEM132B, TMEM245, ZMAT1, ZMYND12, KIAA0930, TCP11L1, TMEM51, VMO1, ARMCX4*

## Results

### Identification of Genes Related to Differentiation Status in Dedifferentiated Thyroid Cancer

**Figure 1:**
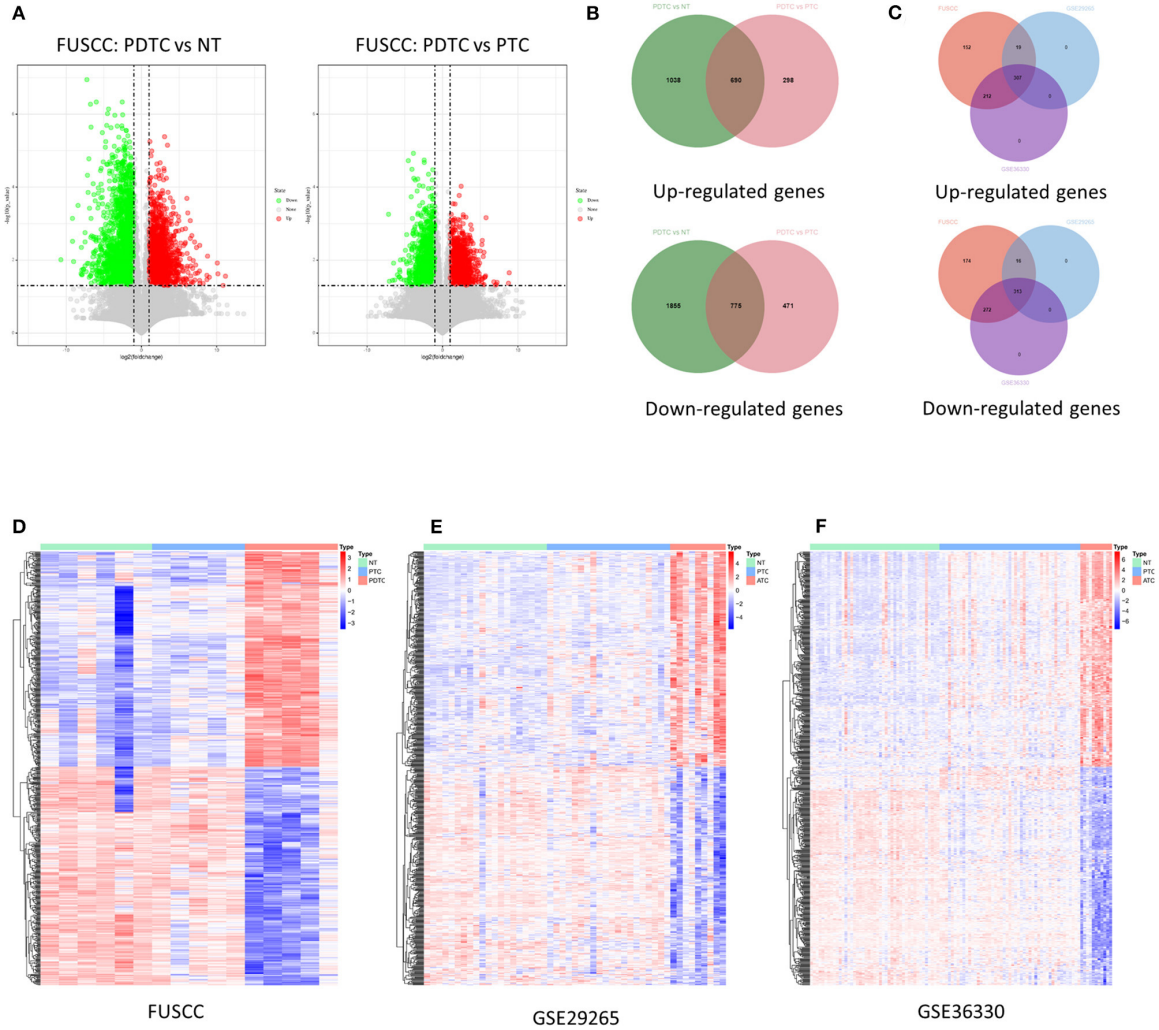
Identification of deregulation of genes in DDTC. **(A)** Volcano maps: left map, 1,728 downregulated genes (green dots) and 988 upregulated genes (red dots) in PDTCs compared with NT tissues; right map, 2,630 downregulated genes (green dots) and 1,246 upregulated genes (red dots) in PDTCs compared with PTCs; fold change (FC) ≥ 2, *p* < 0.05. **(B)** Compared with PTC and NTs, overlap of abnormal expressed genes in PDTC. **(C)** The Venn diagram showed that 1,465 genes abnormally expressed in the FUSCC cohort were validated into GSE29265 and GSE33630, and 313 downregulated genes and 307 upregulated genes were screened out. **(D–F)** The heat map shows the expression data of 620 dysregulated genes in the FUSCC cohort, GSE29265 cohort, and GSE33630 cohort. DDTC, dedifferentiated thyroid cancer; PDTC, poorly differentiated thyroid cancer; NT, normal thyroid; PTC, papillary thyroid cancer; FUSCC, Fudan University Shanghai Cancer Center.

To identify genes that may relate to dedifferentiation of DTC, we first analyzed changes in high-throughput transcriptome expression profile of five PDTCs, five PTCs, and six NTs from the FUSCC cohort. Then, 1,465 deregulated genes (690 upregulated and 775 downregulated, fold change ≥ 2, *p* < 0.05) were found among three groups (Figure 1A). Then we found out the corresponding expression values of these genes in two GEO datasets (GSE29265 and GSE33630) and confirmed their differential expression (307 upregulated genes and 313 downregulated genes, fold change ≥ 1, *p* < 0.05), as shown in Venn diagrams and heat maps, which finally validated our target gene set for an in-depth study (Figures 1B–F).

### Weighted Gene Co-expression Network Analysis Obtained a Co-expression Module Containing Seven Hub Genes

**Figure 2:**
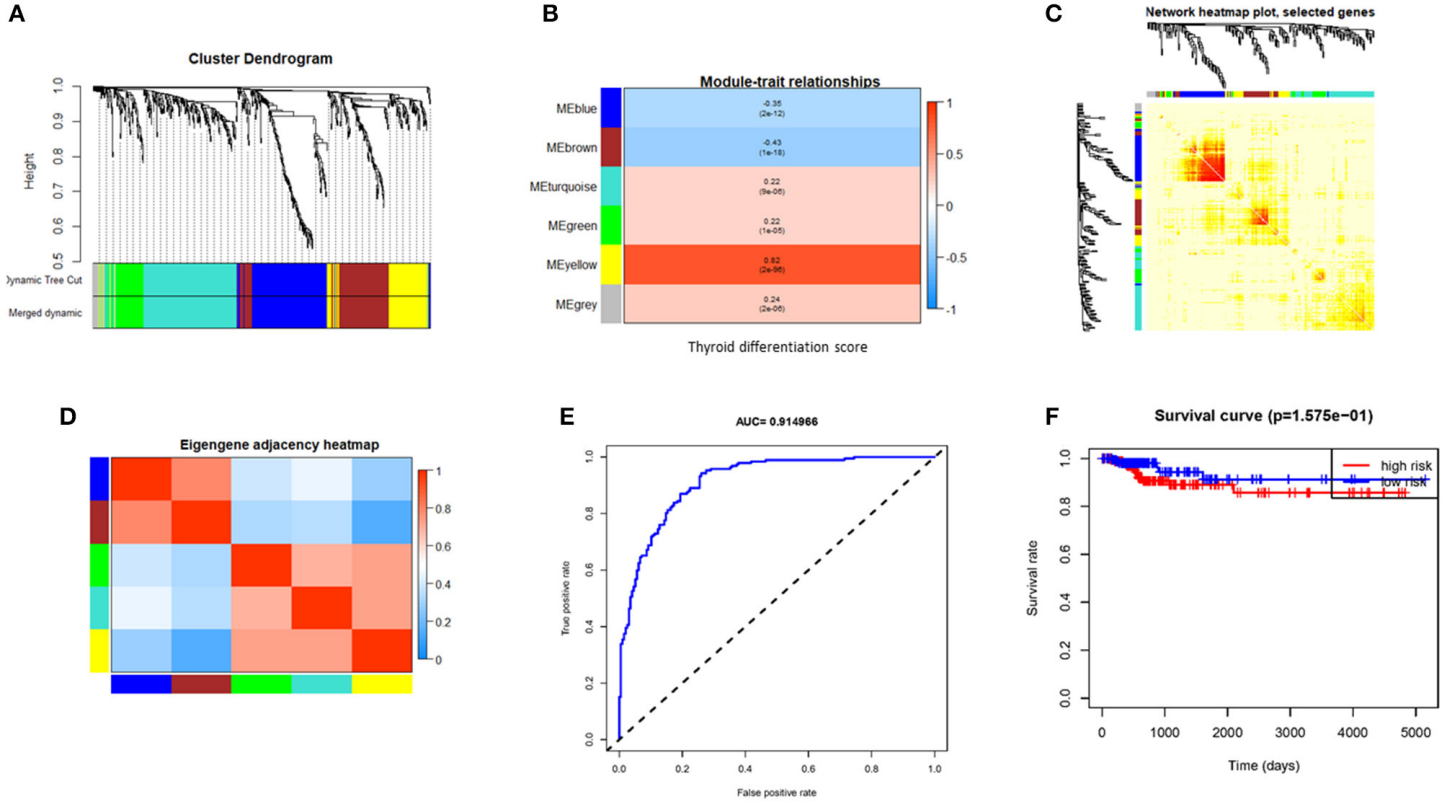
Gene modules identified by weighted gene co-expression network analysis (WGCNA) and indicative ability of hub gene signature constructed by logistic regression for PTC differentiation and disease free survival rate. **(A)** Cluster the gene dendrograms according to topological overlap and assign different colors to each module. Finally, seven co-expression modules were constructed. The size of these modules varies according to the number of genes they contain. **(B)** Module–TDS associations. Each row corresponds to a module eigengene; each column corresponds to TDS. Each cell contains the corresponding correlation and *p*-value. **(C)** Using heat map plots to visualize gene networks. The heat map depicts the topological overlap matrix (TOM) between all genes in the analysis. **(D)** Heat map of module adjacency. Red indicates high adjacency (positive correlation), and blue indicates low adjacency (negative correlation). **(E)** The receiver operating characteristic (ROC) shows indicative ability of hub gene signature for TDS. **(F)** Hub gene signature fails to distinguish DFS well. PTC, papillary thyroid cancer; TDS, thyroid differentiation score; DFS, disease-free survival.

The WGCNA of 620 valid genes was carried out. Seven gene co-expression modules were detected. WGCNA assigned colors to name each module, and the yellow module showed the highest correlation with TDS (Figures 2A–D). Then we screened genes by direct correlation between genes and specified traits, module identity, and weighted correlation; *METTL7A, KCNQ1, ALDH9A1, C16orf46, PLAUR, BCL2*, and *TPMT* were defined as the hub genes. The expression levels of these seven hub genes in each patient were fitted to a dedifferentiation risk score through logistic regression. The AUC value of this risk score in TCGA cohort reached 0.91(Figure 2F) and 0.73 in GEO combined cohort (Supplementary Figure 2). However, after the patients were divided into two groups based on the risk score, the difference in DFS between two groups was not significant (Figure 2E).

### Functional Classification of Differentially Expressed Genes

The seven hub genes obtained by WGCNA showed no good results in functional enrichment and thus cannot fully reflect the impact of abnormally expressed genes on differentiation. Therefore, we screened 620 genes through univariate logistic regression analysis and found 396 genes that significantly correlated with TDS. Next, we tried to group these genes into functional groups based on the results of GSEA. We found that the results of GSEA were mostly concentrated in proliferation, immunity, metabolism, and other related pathways (Supplementary Tables 2, 3). The most significant phenotype of tumor cells after dedifferentiation was the proliferation ability (27, 28); as a result, some phenotypes of interest may have been masked by proliferation during GSEA.

Therefore, we combined the results of GSEA and data from the literature to divide all deregulated genes into 11 groups including cell differentiation and rhythm, substance and signal transduction, transcription and epigenetic modification, protein modification, cilia formation and movement, extracellular matrix (ECM), apoptosis, proliferation, invasion, metabolism, and immunity (Table 2). In addition, there were 44 genes that cannot be clearly functionally classified (Table 1; Supplementary Table 4).

**Table 2:** Risk score for each functional group.

**Group**	**Risk score**
Cell differentiation and rhythm	Risk score = (0.006 × *HHEX*) + (0.057 × *CIPC*) + (−0.381 × *NFIA*) + (−0.506 × *TCTN1*) + (0.056 × *ARNTL2*)
Substance and signal transduction	Risk score = (−0.497 × *KLHDC1*) + (−0.006 × *PEBP1*) + (−0.363 × *FZD5*) + (−0.106 × *KCNQ1*) + (0.048 × *SLC48A1*) + (0.529 × *ILDR1*) + (−0.729 × *ANK3*) + (−0.248 × *TOM1L2*)
Transcription and epigenetic modification	Risk score = (−0.079 × *TMPT*) + (0.087 × *ZSCAN18*) + (−0.405 × *SMAD6*) + (−0.660 × *PRDM16*) + (0.029 × *TBX3*)
Protein modification	Risk score = (−0.091 × *USP54*) + (−0.194 × *RNF180*) + (−0.251 × *ZNF10*)
Cilia formation and movement	Risk score = (−0.854 × *TAPT1*) + (−9.068 × *DNAH7*) + (3.525 × *BBS1*)
Extracellular matrix	Risk score = (−0.200 × *SH3PXD2A*) + (0.112 × *SLC8A1*) + (−0.056 × *CPQ*) + (−0.067 × *PEF1*) + (0.023 × *TNC*)
Apoptosis	Risk score = (0.721 × *TMEM192*) + (−0.412 × *ARHGEF5*) + (−0.381 × *BCL2*) + (0.240 × *SLC20A1*)
Proliferation	Risk score = (0.245 × *CLIP2*) + (−0.724 × *NEK11*) + (0.648 × *NTMT1*) + (4.155 × *IQGAP3*)
Invasion	Risk score = (1.278 × *NHSL1*) + (0.327 × *OCLN*) + (−0.075 × *EPB41L4B*) + (0.096 × *PLAUR*) + (0.201 × *PIK3CB*) + (−0.192 × *EPB41L5*) + (−0.019 × *ID4*) + (−0.050 × *TPM4*) + (−0.525 × *MARVELD2*)
Metabolism	Risk score = (0.131 × *CHPT1*) + (−0.054 × *PDE8B*) + (−0.110 × *HSD17B8*) + (−1.226 × *ADAMTSL1*) + (−0.052 × *ST3GAL1*) + (0.074 × *SLC7A5*) + (0.900 × *ACOT7*) + (−0.225 × *PYGL*)
Immunity	Risk score = (0.059 × *SECTM1*) + (−0.125 × *RAP2B*) + (0.121 × *ELF4*) + (0.131 × *CRBN*) + (−0.140 × *NFKBIE*) + (−0.030 × *SASH1*) + (1.109 × *CEACAM4*) + (0.032 × *OSCAR*)
Ungrouped	–

### Gene Signatures of Signal and Substance Transport, Transcription and Epigenetic Modification, Extracellular Matrix, and Metabolism Group Can Indicate Dedifferentiation of Papillary Thyroid Cancer

Among the 396 genes obtained in multivariate regression analysis, many genes have been extensively studied. For example, *EZH2*, a methyltransferase, is closely related to tumor metastasis and proliferation (29, 30). *TNC*, an extracellular matrix protein, plays an important role in tumor cell invasion and metastasis (31, 32). *PRDM16*, which also plays an important role in protein modification, is also closely related to fat metabolism and tumor growth (33, 34). In addition, there are many important tumor-related genes. We have further screened them in each group to construct a functionally related gene signature.

**Figure 3:**
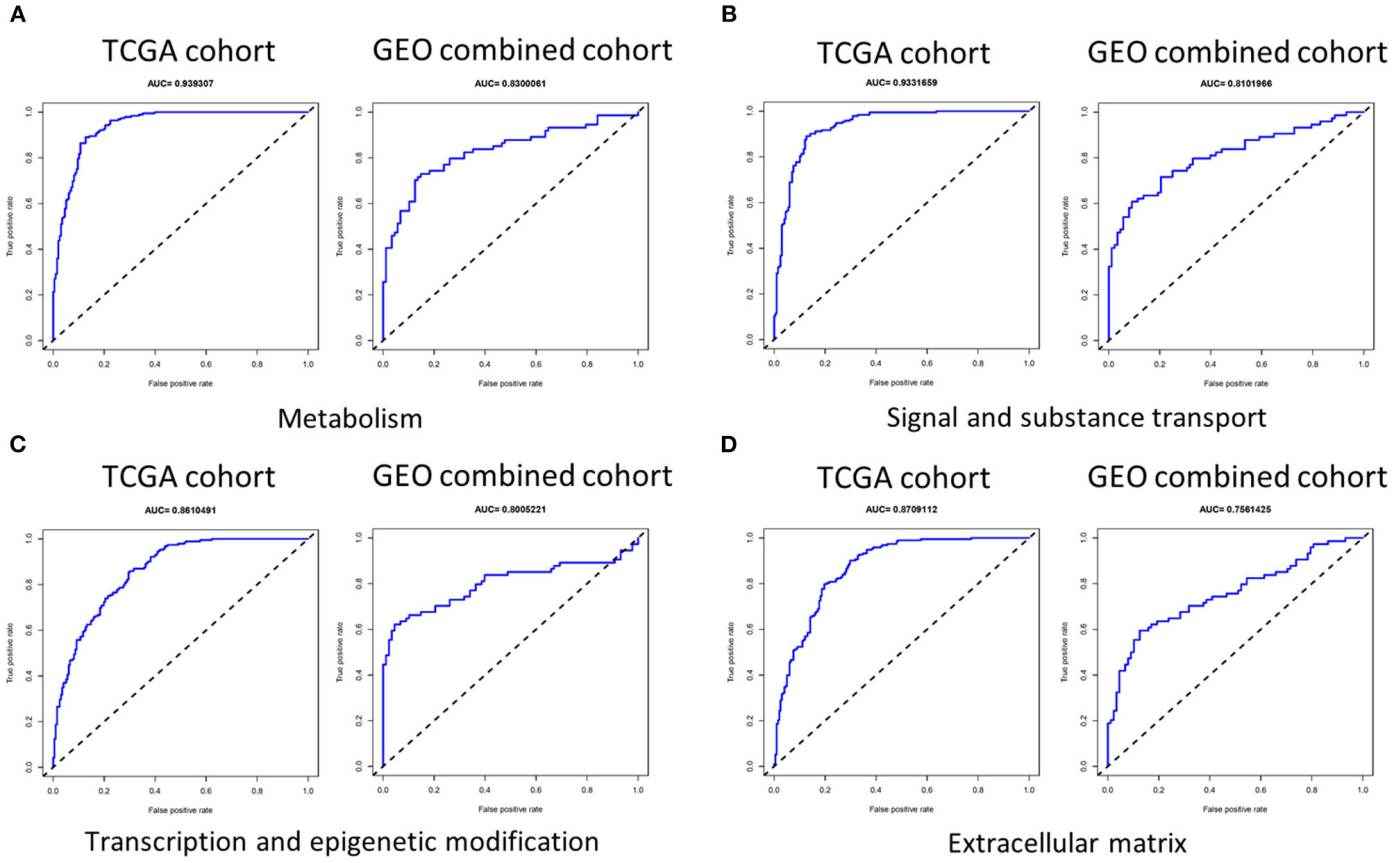
The AUC of four signatures of metabolism, signal and substance transport, transcription and epigenetic modification, and extracellular matrix. **(A–D)** The AUC of the group metabolism, signal and substance transport, transcription and epigenetic modification, and extracellular matrix was >0.75 in both the training set and validation set. AUC, area under the receiver operating characteristic curve.

We first used LASSO regression to screen each group of genes (Supplementary Figure 3) and then performed multivariate logistic regression analysis adjusted by T stage, lymph node metastasis (LNM), and *BRAF*^*V*600*E*^ on each group of genes selected to determine genes to be incorporated into the signature, and we evaluated the indicative ability of each signature by calculating the AUC value in the training set (TCGA cohort) and validation set (GEO combined cohort). We found that the AUC value in the group signal and substance transport, transcription and epigenetic modification, ECM, and metabolism was >0.75 in both the training set and validation set (Figure 3). It was worth noting that in two datasets, the AUC values of the invasion group reached 0.919297 and 0.746622, and the cilia formation and movement group also reached 0.805777 and 0.709767 (Supplementary Figure 4).

### Correlations of the Gene Signatures With Clinical Features of Papillary Thyroid Cancer

In further analyses, we investigated the clinicopathological significance of the gene signatures associated with dedifferentiation to reveal their further research priority and translational potential.

**Figure 4:**
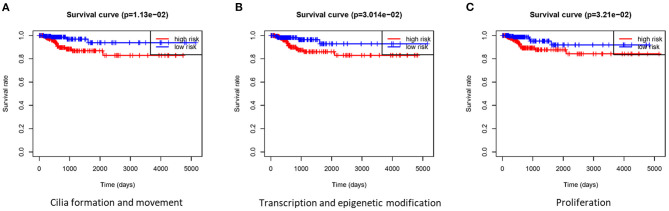
Gene signatures were tested for DFS analyses in patients with thyroid cancer. **(A–C)** There were significant differences in DFS between the high-risk group and low-risk group in cilia formation and movement, transcription and epigenetic modification, and proliferation groups. DFS, disease-free survival.

**Figure 5:**
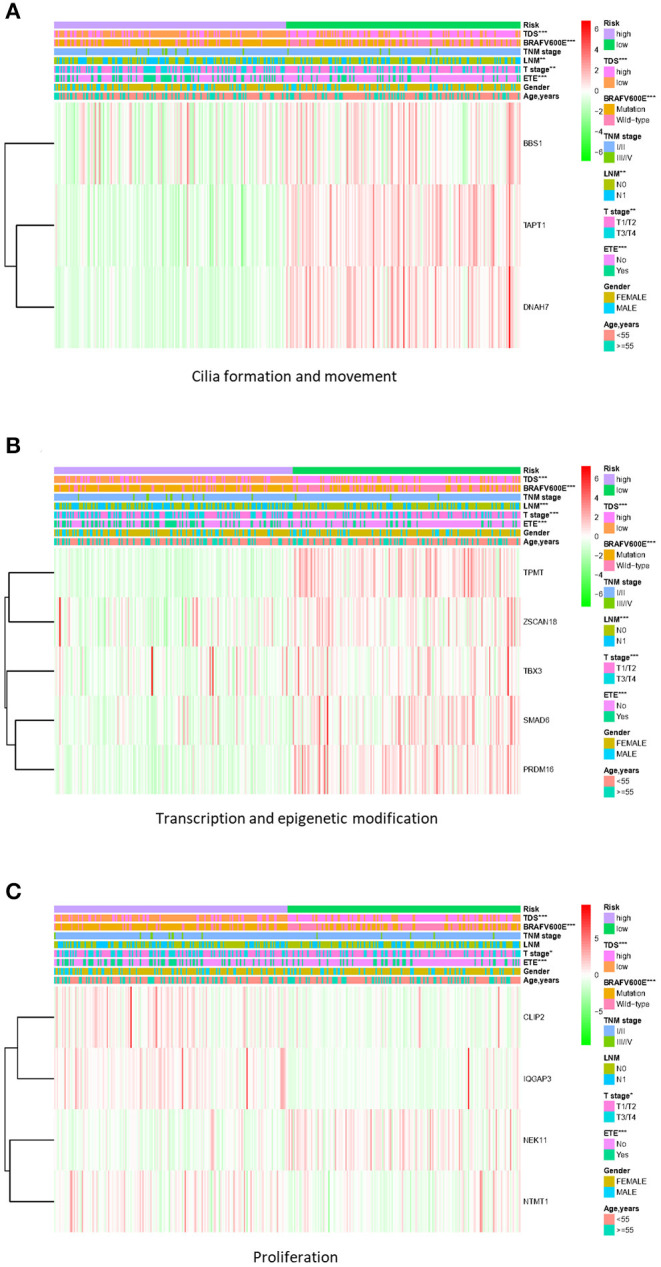
Correlation of risk score with clinical parameters in TCGA cohort. **(A)** The risk score in cilia formation and movement group was significantly correlated with TDS, *BRAF*^*V*600*E*^ mutation, LNM, T stage, and extrathyroidal extension (ETE). **(B)** The risk score in the transcription and epigenetic modification group was significantly correlated with TDS, *BRAF*^*V*600*E*^ mutation, LNM, T stage, and ETE. **(C)** The risk score in the proliferation group was significantly correlated with TDS, *BRAF*^*V*600*E*^ mutation, T stage, and ETE. *p < 0.05, **p < 0.01, and ***p < 0.001. TCGA, The Cancer Genome Atlas; TDS, thyroid differentiation score; LNM, lymph node metastasis.

Based on the gene signature of each group, we divided all TCGA patients into high-risk and low-risk groups, and we analyzed their survival differences. We found that although some risk score of gene signatures can reflect degree of differentiation well, they cannot accurately reflect the patient's tumor recurrence status. Among all gene signatures, there were significant differences in DFS between the high-risk and low-risk groups including transcription and epigenetic modification, cilia formation and movement, and proliferation (Figure 4; Supplementary Figure 5). Next, we further analyzed the relationships between high or low risk and clinical parameters in TCGA cohort (Table 3), and we found significant differences in *BRAF*^*V*600*E*^ mutation, TNM stage, T stage, and TDS. In addition, in the cilia formation group, the risk score and LNM also had a significant correlation (Figure 5).

**Table 3:** Clinicopathologics of PTC patients in TCGA cohort.

	**TCGA (*N* = 354)**	
**Variables**	***N***	**%**
Age, years, mean ± SD (range)	47.53 ± 15.79 (15–89)	
**Gender**		
Male	91	25.71
Female	263	74.29
**ETE**		
Yes	100	28.25
No	254	71.75
**T stage**		
T1/T2	229	64.69
T3/T4	125	35.31
**LNM**		
N0	208	58.76
N1	146	41.24
**TNM stage**		
I/II	340	96.05
III/IV	14	3.95
***BRAF*^*V*600*E*^**		
Yes	210	59.32
No	144	40.68
**TDS**		
Low	177	50.00
High	177	50.00

*PTC, papillary thyroid cancer; TCGA, The Cancer Genome Atlas; ETE, extrathyroidal extension; LNM, lymph node metastasis*.

## Discussion

Undifferentiated thyroid cancer is relatively rare, but it has a very high degree of malignancy, which brings great difficulties in exploring its pathogenesis. In recent years, many studies have explored undifferentiated thyroid cancer at pre-transcription, transcription, and translation levels, revealing that a considerable number of undifferentiated cancers develop from dedifferentiation of DTC. Studies have shown that dedifferentiation of colorectal cancer is closely related to TGF-β, Wnt, and Hedgehog signaling pathways (35), while dedifferentiation of glioblastoma is closely related to hypoxia (36). Our previous studies have also shown that metabolic pathways play an important role in dedifferentiation of thyroid cancer (13). Most of these studies focused on a certain pathway or mechanism; however, according to previous experience, the occurrence and development of malignant tumors, including dedifferentiation, involve multiple mechanisms and a large number of abnormal gene expressions. Moreover, research on the mechanism of dedifferentiation of DTC is still not comprehensive. In order to explore the potential mechanism of dedifferentiation of DTC from a broader perspective, we retrospectively obtained 16 samples of eight patients with DDTC who had undergone surgical treatment in our institution (FUSCC cohort). We used intermittent sampling to obtain tissue samples with a gradient of differentiation. Through the joint analysis of 16 samples of high-throughput sequencing data, TCGA cohort, and GEO chip data, we obtained 620 differentially expressed genes related to dedifferentiation of PTC, and all of them were subjected to further analysis.

First, we performed WGCNA on all 620 genes and divided them into seven modules according to their expression. Among them, the yellow module demonstrated the strongest correlation with TDS. After screening genes by direct correlation between genes and specified traits, module identity, and weighted correlation, seven genes including *METTL7A, KCNQ1, ALDH9A1, C16orf46, PLAUR, BCL2*, and *TMPT* have been certified as hub genes. Through analysis of GeneMANIA prediction server, we found that these seven hub genes and their interacting genes directly or indirectly interact and co-localize, but they were not functionally enriched together (Supplementary Figure 6). Thus, we believe that the results of WGCNA cannot fully explain the dedifferentiation process of DTC.

In order to reflect the differentiation status more comprehensively from multiple angles, we first performed GSEA on all genes and found that the pathways related to TDS were mostly enriched on proliferation and apoptosis, which is consistent with the biological performance of PDTC. Many studies also focused on pathways related to cell proliferation, invasion, and immunity (26, 37–39); but also many new tumor-related phenotypes, such as ECM, cilia formation and movement, and epigenetic modification, have attracted our interest (40–43). However, these phenotypes have been rarely studied in thyroid cancer, so there is a certain value for further research (9, 44, 45).

We evaluated the gene signature of each group by calculating AUC, and we verified it in the combined GEO cohort. Finally, we identified gene signatures in group signal and substance transport, transcription and epigenetic modification, ECM, invasion, and metabolism groups, which showed very good indicative performance. In the gene signature of signal and substance transport group, *PEBP1, FZD5, KCNQ1*, and *TOM1L2* play a role in protein kinase binding together (Supplementary Figure 7A), while *PEBP1* is also associated with autophagy and ferroptotic death (46). In the transcription and epigenetic modification group, *SMAD6* and *PRDM16* showed a high degree of consistency in function (Supplementary Figure 7B), as functions of these two genes are concentrated on TGF-β/SMAD (47, 48), a pathway that plays an important role in cell differentiation. *SMAD6* is a component of this pathway; and its importance is self-evident, as *PRDM16*, an important methyltransferase and transcription factor, has also been reported as a repressor of the TGF-β/SMAD pathway (49). In the ECM group, *TNC* was in a central position, and its main interaction target was Integrin Alpha V (*ITGAV*), a protein expressed in thyroid tissues higher than in other tissues in the human body, suggesting that it specifically influences the differentiation of thyroid cancer through the phenotype of extracellular matrix (Supplementary Figure 7C). As a traditional malignant tumor phenotype, the gene signature of the invasion group consisted of *NHSL1, OCLN, EPB41L4B, PLAUR, PIK3CB, EPB41L5, ID4, TPM4*, and *MARVELD2*. *TPM4* showed the opposite effects in colon cancer and lung cancer. In our analysis, its high expression corresponded to better differentiation, which is consistent with the research results in colon cancer (50, 51). In the metabolism group, *CHPT1, PDE8B, HSD17B8, ADAMTSL1, ST3GAL1, SLC7A5, ACOT7*, and *PYGL* were combined to make up a signature. *PDE8B, HSD17B8, ST3GAL1*, and *ACOT7* also appeared in a signature constructed in our previous study (13).

Through the STRING database, we found that some genes have an interaction relationship, including intra-group and inter-group interactions (52) (Supplementary Figure 8). In the transcription and epigenetic modification group, there are gene fusion and co-expression between *TBX3* and *SMAD6* (53). *OCLN* and *MARVELD2* are both invasion-related genes, and they share a protein homology (54). *MARVELD2* also has a co-expression relationship with the signal and substance transport gene *ILDR2* (54). *KLHDC1* in the signal and substance transport group, and *IQGAP3* in the proliferation group also has co-expression (55). These possible interactions indicate that the joint effect of these genes may have a more important impact on differentiation of DTC. Since they have not been studied in thyroid cancer, it is worthy of further investigation.

At the same time, we also paid due attention to the relationship between risk scores and other clinical parameters. We found that the cilia formation group could better reflect the condition of LNM than other groups. Other studies have shown that cilia formation is closely related to autophagy (56); therefore, high expression of cilia formation-related proteins (*DNAH7, TAPT1*, and *BBS1*) in the low-risk group may be due to exuberant autophagy activity (Supplementary Figure 7D). Additionally, the formation and movement of cilia are also very important to the movement of cells, which in turn affect the tumor cell migration and invasion (57). In recent years, studies have found that the sensitivity of ATC to chemotherapy drugs is closely related to autophagy (58–61); therefore, we believe that genes involved in cilia formation and movement are promising as targets for drug therapy and should be further researched.

Our research also had certain limitations. First, we selected traditional malignant tumor-related phenotypes and some phenotypes that we were interested in as groupings, so this method is not very objective. There may be some important phenotypes that we did not include in the groups. This has also led to 44 genes that could not be included in specific group. At the same time, it should be noted that a more detailed and comprehensive grouping may divide the genes too finely, resulting in some interactions that cannot be reflected in intergroup. Second, limited by the number of cases and difficulty of obtaining material, the distinction between PDTC and PTC in the sequenced samples is not fine enough. We plan to collect more samples and use more precise microdissection methods for the next step of research.

In conclusion, we analyzed the genes that may affect the differentiation of thyroid cancer from 11 different perspectives and constructed gene signatures, revealing the possibility of multiple mechanisms that lead to dedifferentiation of DTC.

## Data Availability Statement

The datasets presented in this study can be found in online repositories. The name of the repository and accession number can be found below: National Center for Biotechnology Information (NCBI) BioProject, https://www.ncbi.nlm.nih.gov/bioproject/, PRJNA702648.

## Ethics Statement

This study was approved by the Medical Ethics Committee of the FUSCC. All procedures performed in our study were in accordance with the ethical standards of our institutional research **c**ommittee and the Declaration of Helsinki.

## Author Contributions

WX, CL, BM, and WW contributed to conception and design of the study. HJ, XW, and YY organized the data. WX, CL, and YucW performed the statistical analysis. All authors contributed to writing and review of the manuscript and approved the final submitted version.

## Conflict of Interest

The authors declare that the research was conducted in the absence of any commercial or financial relationships that could be construed as a potential conflict of interest.
