# Supplementary material for: Identification of Key Functional Gene Signatures Indicative of Dedifferentiation in Papillary Thyroid Cancer
Source: Front Oncol. 2021 Apr 28;11:641851. doi: 10.3389/fonc.2021.641851 (PMC8113627; doi:10.3389/fonc.2021.641851)
Supplement: Supplementary file 1 [file Table_1.DOC]

**Table S1. The information of patients and samples from the FUSCC**

| **Case ID** | **Age (years)** | **Sex** | **Histopahtology** | **Sample types** | **RNA sequencing** | ***BRAF^V600E^*** | ***RAS*** | ***TERT*** | ***TP53*** | ***PIK3CA/AKT/mTOR*** | **Other genetic events** |
| --- | --- | --- | --- | --- | --- | --- | --- | --- | --- | --- | --- |
| Case 1 | 85 | Male | PDTC | FFT | Yes | Yes | No | Yes | Yes | No | *MLH1/KIT/MET* |
|  |  |  | PTC | FFT | Yes | Yes | No | Yes | No | No | *MLH1/KIT* |
|  |  |  | NT | FFT | Yes | No | No | No | No | No |  |
| Case 2 | 48 | Male | PDTC | FFT | Yes | No | Yes (*HRAS*) | Yes | No | No | *MET/APC* |
|  |  |  | PTC | FFT | Yes | No | Yes (*HRAS*) | No | No | No | *MET/APC* |
|  |  |  | NT | FFT | Yes | No | No | No | No | No |  |
| Case 3 | 64 | Male | PDTC | FFT | Yes | No | No | No | Yes | No | *APC* |
|  |  |  | NT | FFT | Yes | No | No | No | No | No |  |
| Case 4 | 70 | Male | PDTC | FFT | Yes | Yes | No | No | Yes | Yes (*PIK3CA*) | *PDGFRA* |
|  |  |  | NT | FFT | Yes | No | No | No | No | No |  |
| Case 5 | 42 | Male | PTC | FFT | Yes | Yes | No | NA | NA | NA | NA |
|  |  |  | NT | FFT | Yes | No | No | No | No | No |  |
| Case 6 | 39 | Female | PTC | FFT | Yes | NA | NA | NA | NA | NA | NA |
|  |  |  | NT | FFT | Yes | No | No | No | No | No |  |
| Case 7 | 73 | Female | PDTC | FFT | Yes | NA | Yes (*NRAS*) | Yes | Yes | No | *APC/KIT/PDGFRA* |
| Case 8 | 60 | Male | PTC | FFT | Yes | NA | NA | NA | NA | NA |  |

Abbreviations: FUSCC, Fudan University Shanghai Cancer Center; PDTC, poorly differentiated thyroid cancer; FFT, fresh frozen tissues; PTC, papillary thyroid cancer; NA, not available.
