# Supplementary figures and images for: Identification of Key Functional Gene Signatures Indicative of Dedifferentiation in Papillary Thyroid Cancer
Source: Front Oncol. 2021 Apr 28;11:641851. doi: 10.3389/fonc.2021.641851 (PMC8113627; doi:10.3389/fonc.2021.641851)

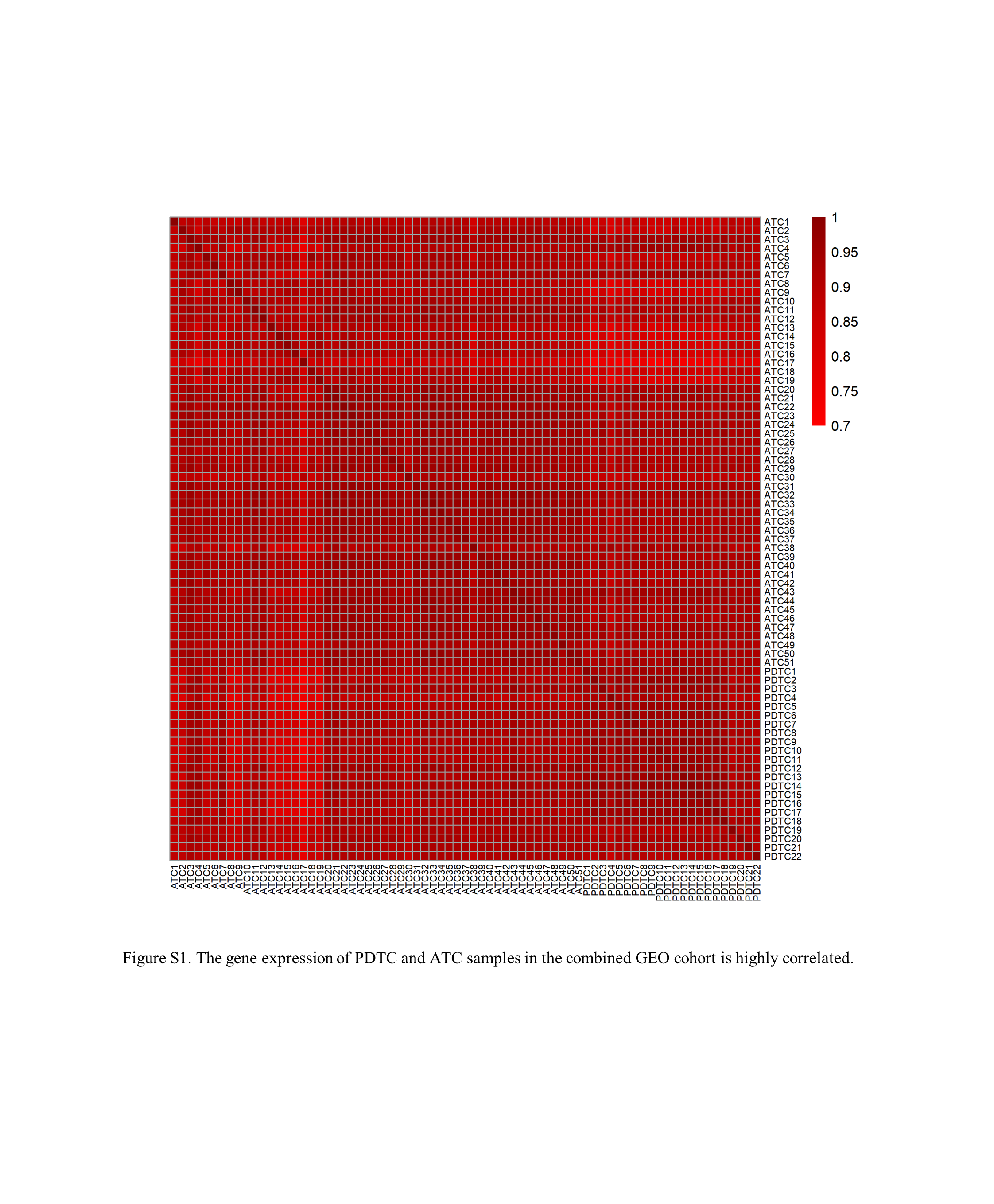

Supplement: Supplementary file 5 [file Image_1.TIF]

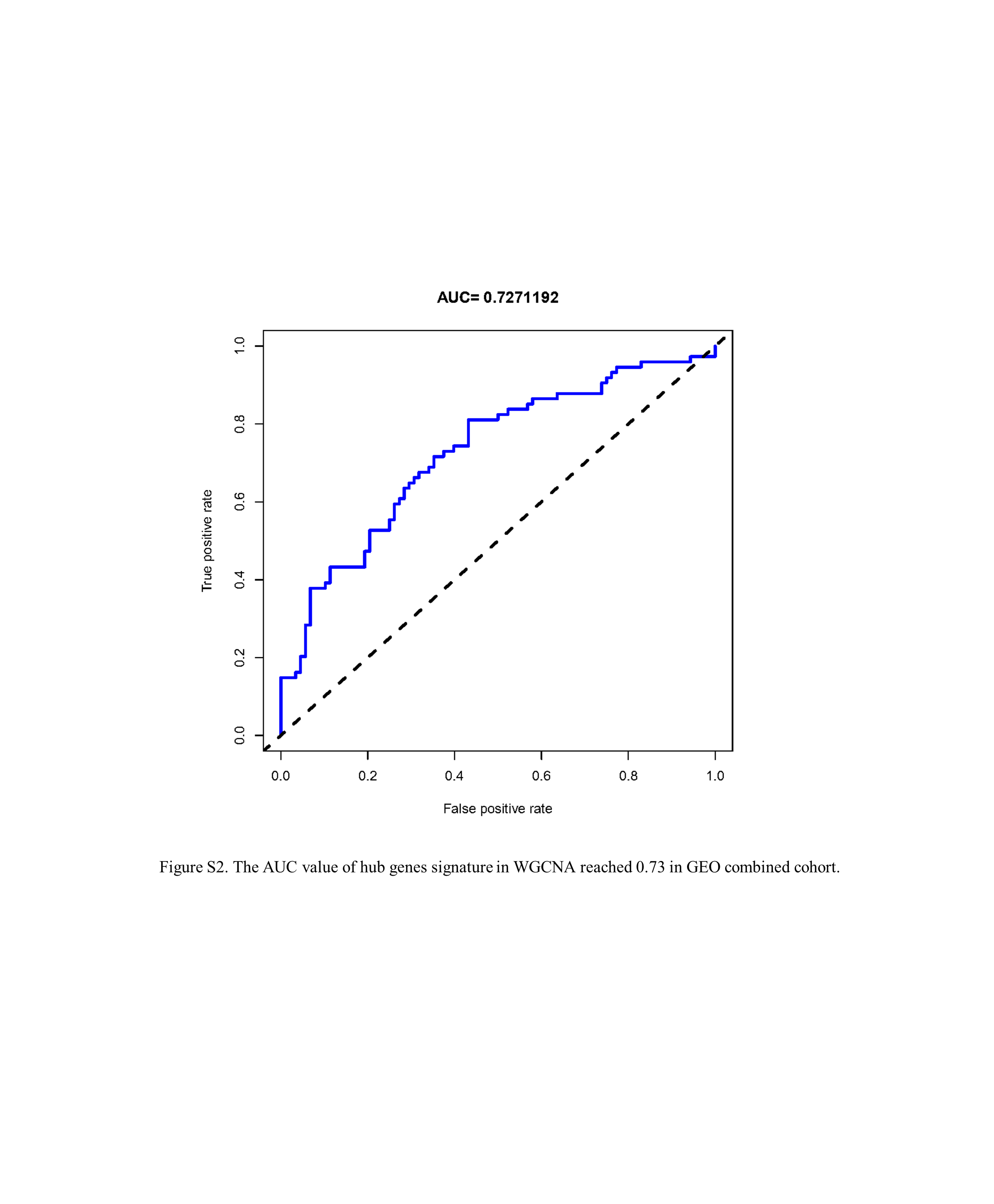

Supplement: Supplementary file 6 [file Image_2.TIF]

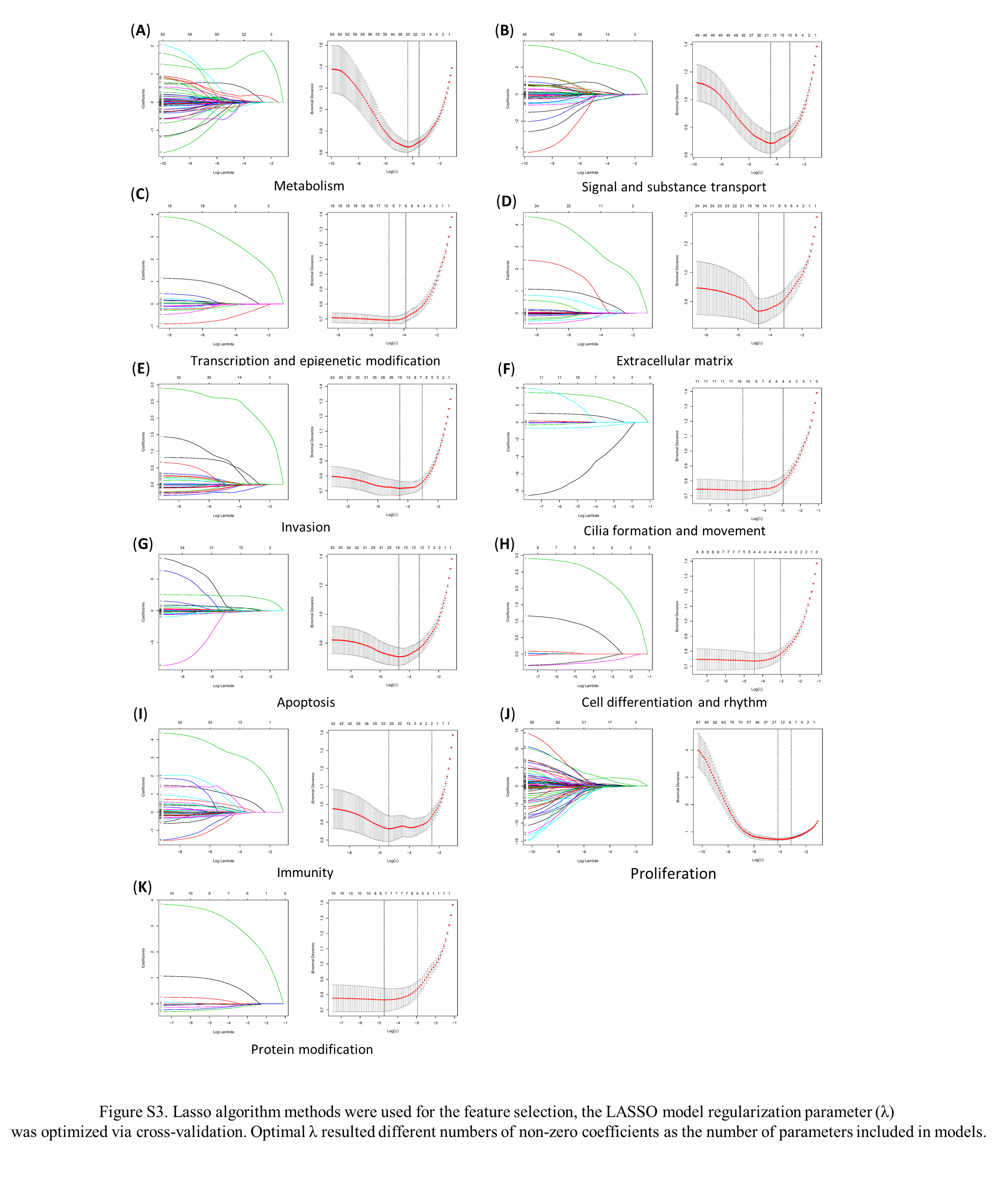

Supplement: Supplementary file 7 [file Image_3.TIF]

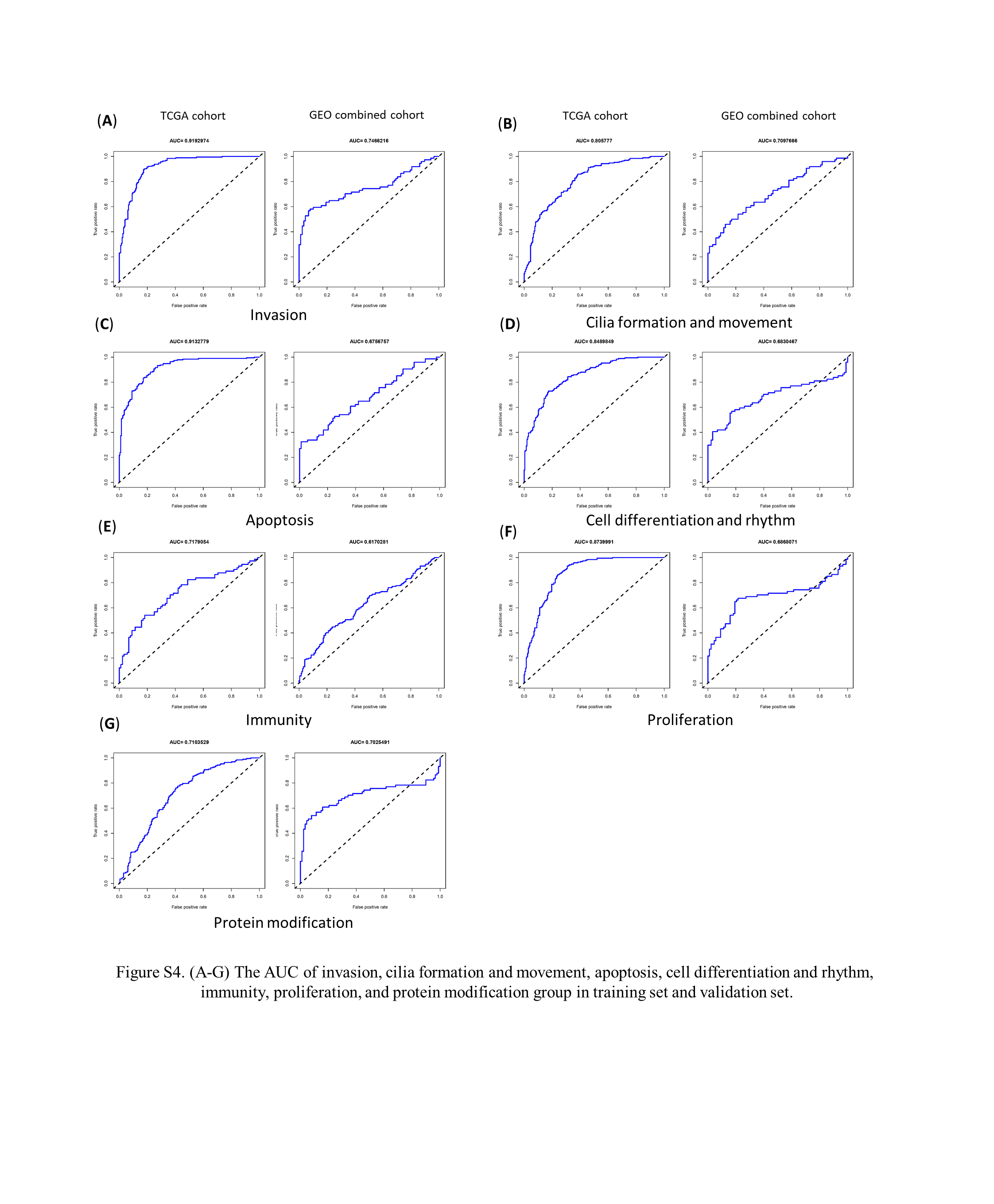

Supplement: Supplementary file 8 [file Image_4.TIF]

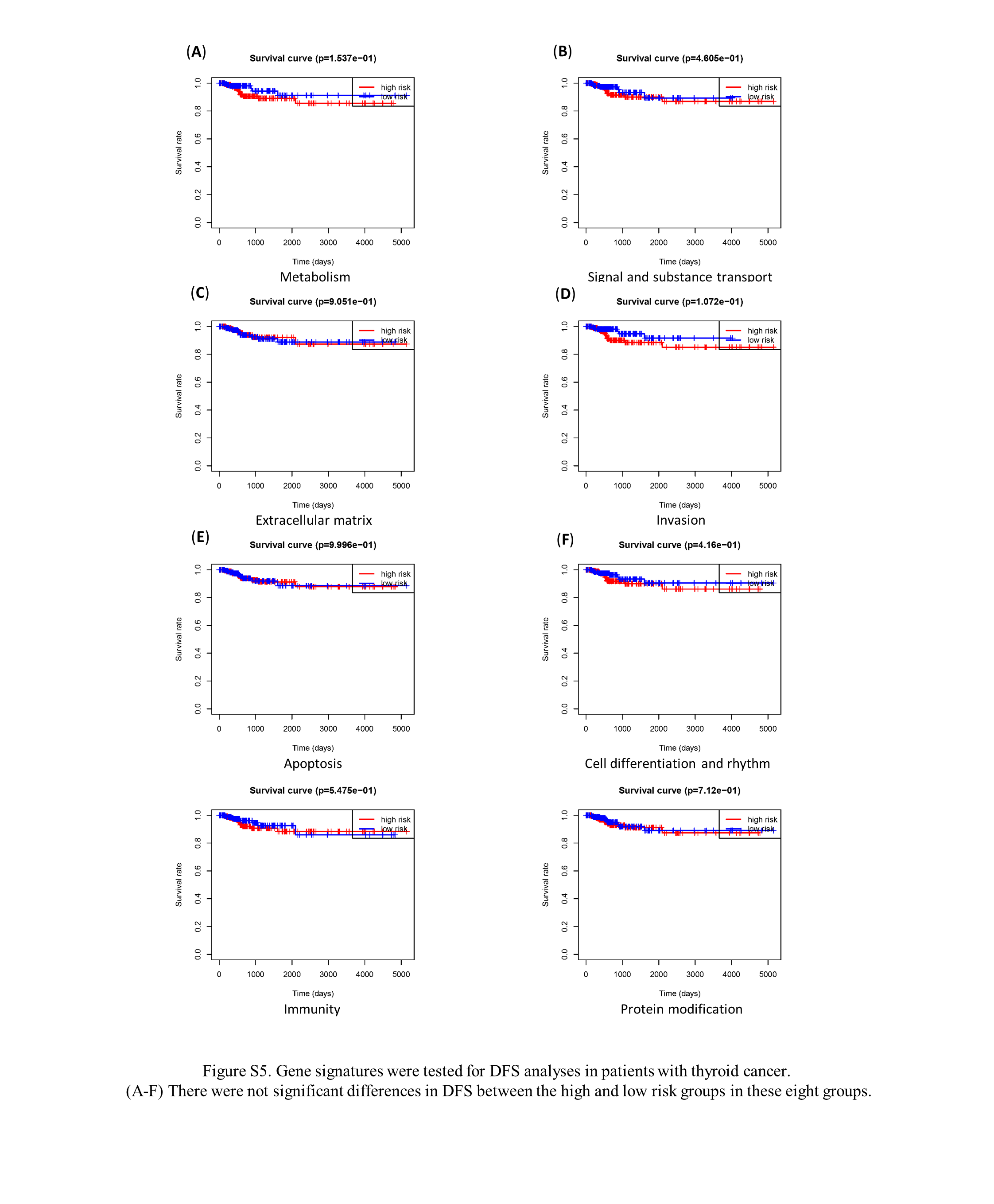

Supplement: Supplementary file 9 [file Image_5.TIF]

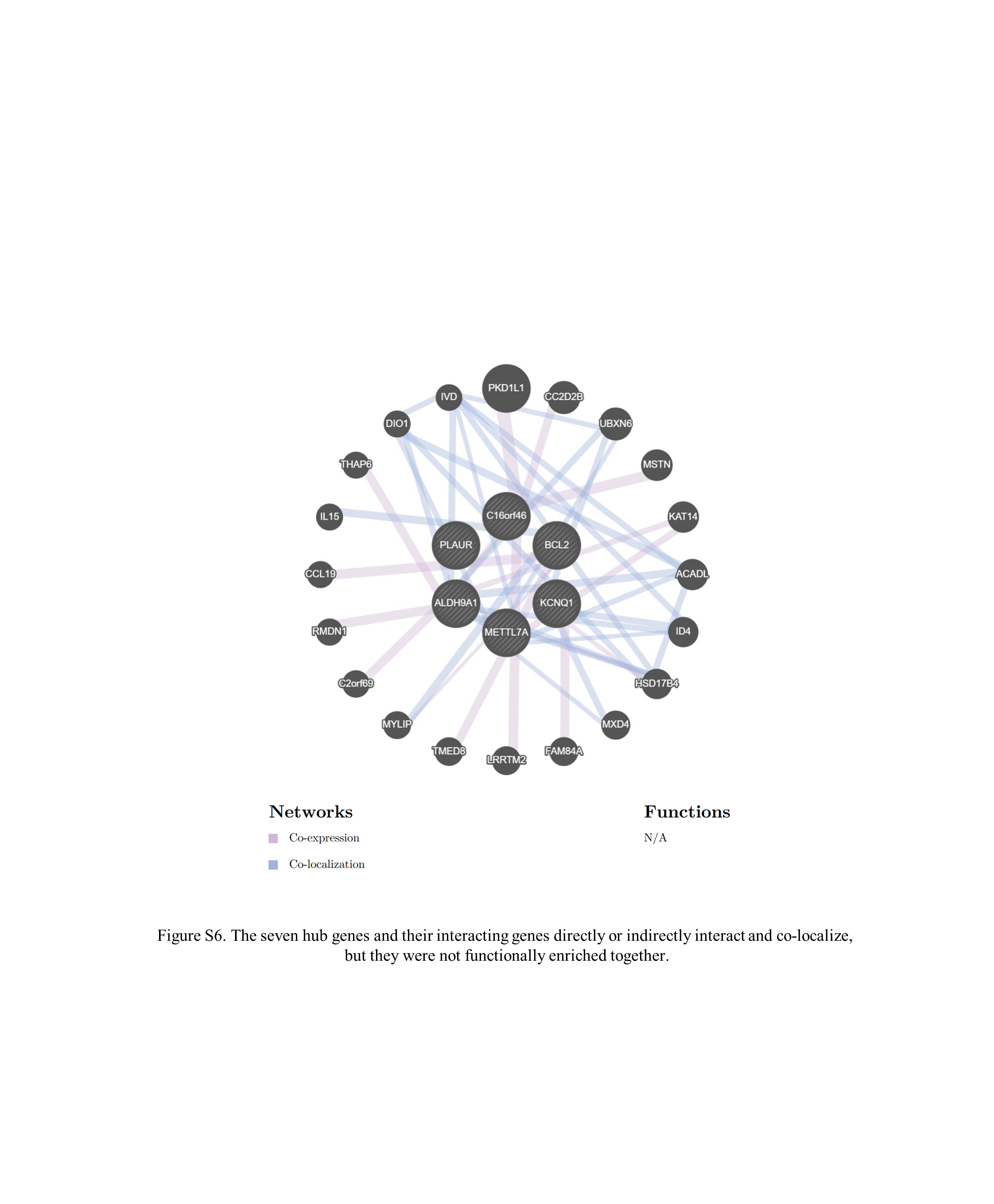

Supplement: Supplementary file 10 [file Image_6.TIF]

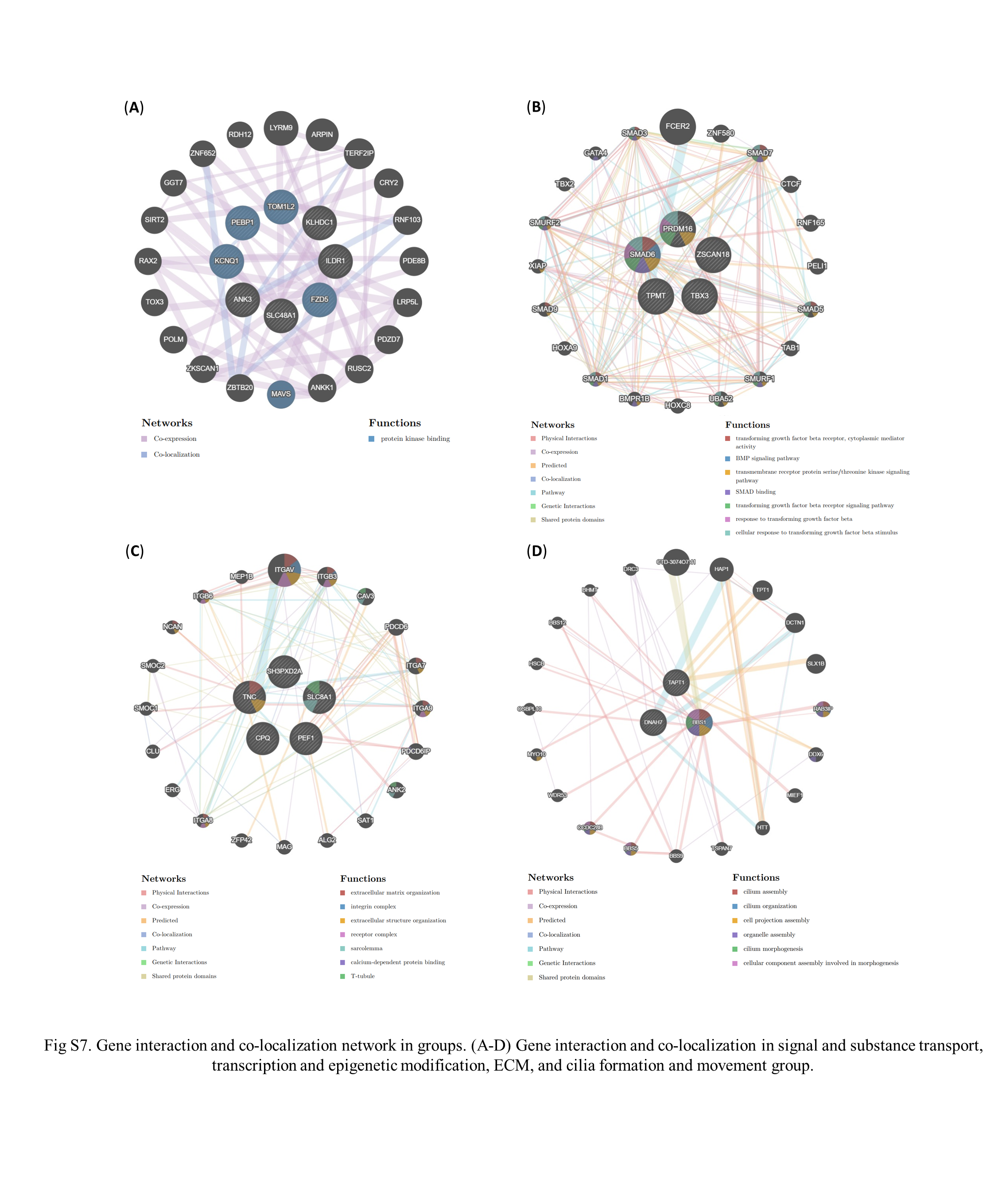

Supplement: Supplementary file 11 [file Image_7.TIF]

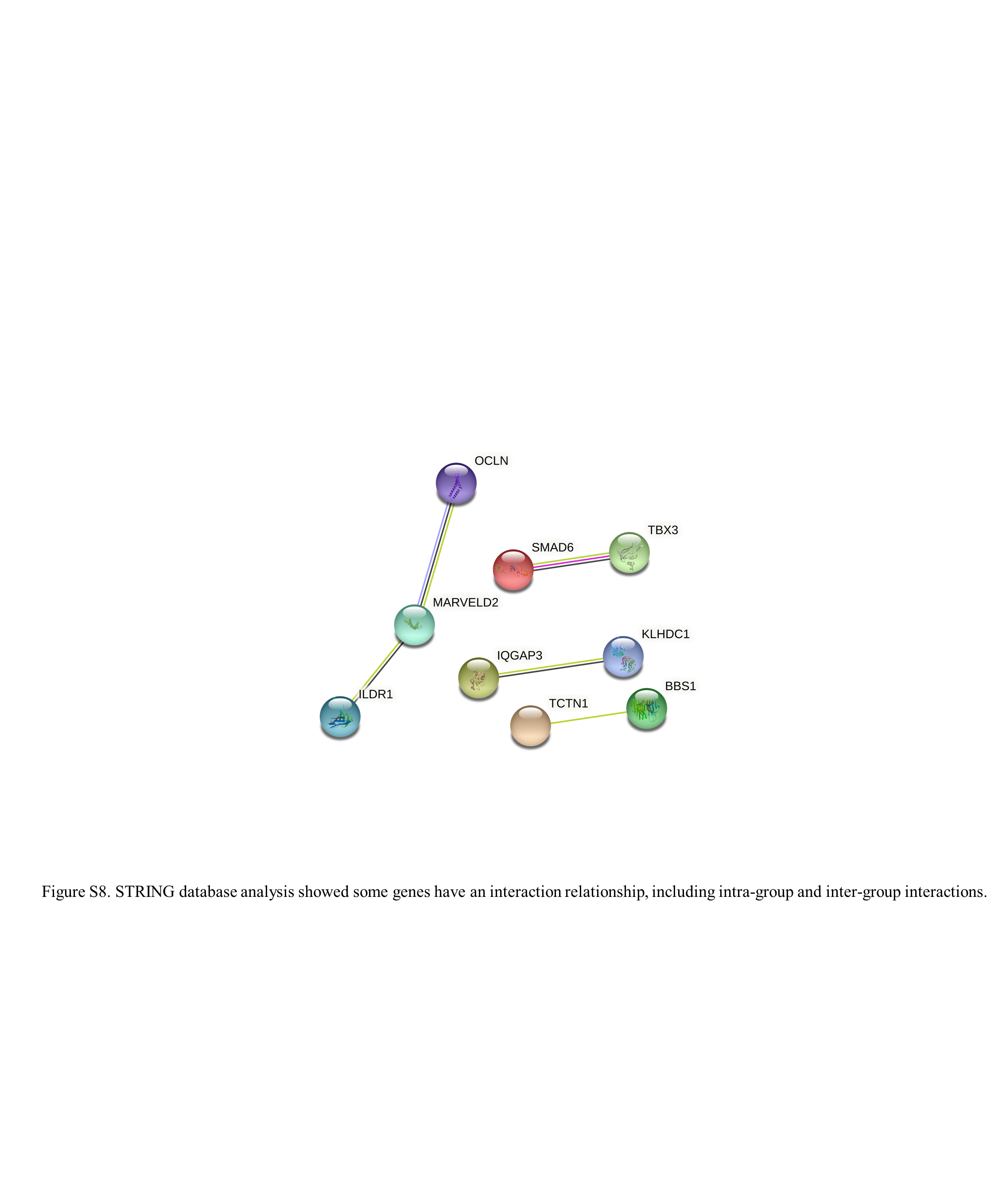

Supplement: Supplementary file 12 [file Image_8.TIF]
